# A correlated motif approach for finding short linear motifs from protein interaction networks

**DOI:** 10.1186/1471-2105-7-502

**Published:** 2006-11-16

**Authors:** Soon-Heng Tan, Willy Hugo, Wing-Kin Sung, See-Kiong Ng

**Affiliations:** 1 Knowledge Discovery Department, Institute for Infocomm Research, Singapore; 2 Department of Computer Science, National University of Singapore, Singapore; 3 Department of Information and Mathematical Science, Genome Institute of Singapore; 4 Present address: Department of Medical Genetics and Microbiology, University of Toronto, Toronto, Canada

## Abstract

**Background:**

An important class of interaction switches for biological circuits and disease pathways are short binding motifs. However, the biological experiments to find these binding motifs are often laborious and expensive. With the availability of protein interaction data, novel binding motifs can be discovered computationally: by applying standard motif extracting algorithms on protein sequence sets each interacting with either a common protein or a protein group with similar properties. The underlying assumption is that proteins with common interacting partners will share some common binding motifs. Although novel binding motifs have been discovered with such approach, it is not applicable if a protein interacts with very few other proteins or when prior knowledge of protein group is not available or erroneous. Experimental noise in input interaction data can further deteriorate the dismal performance of such approaches.

**Results:**

We propose a novel approach of finding correlated short sequence motifs from protein-protein interaction data to effectively circumvent the above-mentioned limitations. *Correlated motifs *are those motifs that consistently co-occur only in pairs of interacting protein sequences, and could possibly interact with each other directly or indirectly to mediate interactions. We adopted the (*l*, *d*)-motif model and formulate finding the correlated motifs as an (*l*, *d*)-*motif pair finding *problem. We present both an exact algorithm, D-MOTIF, as well as its approximation algorithm, D-STAR to solve this problem. Evaluation on extensive simulated data showed that our approach not only eliminated the need for any prior protein grouping, but is also more robust in extracting motifs from noisy interaction data. Application on two biological datasets (SH3 interaction network and TGF*β *signaling network) demonstrates that the approach can extract correlated motifs that correspond to actual interacting subsequences.

**Conclusion:**

The correlated motif approach outlined in this paper is able to find correlated linear motifs from sparse and noisy interaction data. This, in turn, will expedite the discovery of novel linear binding motifs, and facilitate the studies of biological pathways mediated by them.

## Background

An important class of interaction switches for biological circuits and disease pathways are the binding motifs [1,2]. These are very short, functional regions on the proteins that conform to particular sequence patterns; a well-known example is the set of peptides expressing a PxxP consensus (where *x *represent any arbitrary amino acid) that bind SH3 protein domains [3,4]. Finding such motifs is important for drug discovery as many have been implicated in disease pathways. For instance, the proline-rich motifs and glutamine-rich motifs have been linked to Alzheimer's disease, Muscular Dystrophy [5] and Huntington's disease [6]. Recently, Marti *et. al. *reported that the short linear sequence motif RxLx [QE] played a key role in the pathogenesis of malaria [7,8].

Binding motifs can be discovered by biological experiments, such as *site-directed mutagenesis *and *phage display*, which are laborious and expensive. However, given a set of protein-protein interaction data, binding motifs can be discovered computationally as follows: (i) group protein sequences that interact with the same protein, and (ii) for each set of protein sequences grouped, extract the motifs using motif discovery algorithms like MEME [9], Gibbs Sampler [10], PRATT [11] and TEIRESIAS [12]. For example, to computationally detect any possible motif binds by protein *Crk*, we could input protein sequences interacting with *Crk *to motif discovery programs. The underlying assumption is that *Crk *binds through similar sequence segments in many of its interaction partners, which can be detected by string pattern algorithms. For discussion, we denote such approach as One-To-Many (OTM) since we start with one protein to derive a group of multiple proteins associated with it for motif extraction.

The OTM approach is effective only when the protein we start with have enough number of interacting partners for motif extraction. In reality, many proteins have limited interacting partners [13]. This means that for many of the proteins, the signals from the few and short motif instances would be too weak for detection by the existing motif discovery algorithms. The scenario is actually worse when we further consider the high noise levels in interaction data [14] and the inherent heterogeneity of protein interactions – not all the real interacting partners of a protein necessarily carry the same binding motif. In the extreme cases of proteins having only one known interacting partner, it is impossible to extract binding motifs using the OTM approach.

Sometimes, it is possible to apply some known knowledge of protein groups to increase the number of sequences for motif extraction. For example, if individual copies of the SH3 domain bind limited protein partners, we could pool all sequences that bind any SH3 domain proteins to increase the PxxP motif's instances for its "discovery". We denote this approach as the Many-to-Many (MTM) approach since we derived a set of sequences for motif extraction from another set of sequences (protein group). Reiss and Schwikowski adopted an MTM-based method with a modified Gibbs sampling algorithm to enhance motif finding on proteins with limited binding partners and successfully extracted more motifs than the OTM-based approaches [15]. In another work, Neduva *et. al. *complement the OTM approach with MTM approach to find novel linear motif from protein interaction data [16]. However, the MTM will not be applicable if prior knowledge on the protein group is not available. Even if the knowledge are available, they might be incomplete, erroneous or just too generic. As a result, finding motifs from the interacting partners of such a group might often yield less satisfactory results.

In this paper, we are interested in the case when the linear motif in question actually bind directly or interact indirectly with another linear motif. It makes a lot of sense since linear motifs are in general short enough that most of the time it interacts with a similarly short region on the other protein. For modular interaction domains, for example, it is often the subregions, rather than the entire domains, that are involved in mediating protein-protein interactions. In essence, we are modelling interactions as mediated by pair of motifs each occurring in separate proteins that are interacting, and this work revolves around discovering such motif pairs from protein interaction data.

**Figure 1:**
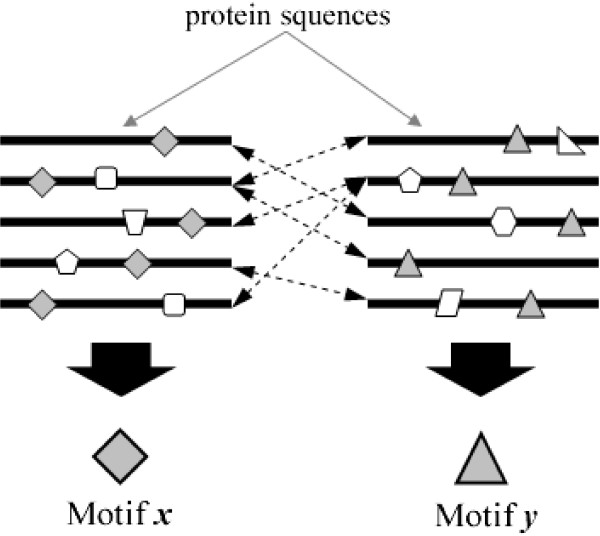
**Correlated motif pair approach**. A depiction of our approach for finding correlated motifs. The dotted lines indicates the interactions between the proteins.

Formally, suppose a set of protein-protein interactions occurring between sequences containing the linear motif *x *and sequences containing the linear motif *y*, we present a novel approach to simultaneously find both motifs *x *and *y *directly from protein interaction data. It is based on the intuition that if a set of interactions were indeed mediated by *x *and *y*, they will be presented for extraction as over-represented co-occurring similar substring pairs found in pairs of interacting proteins in the data set (see Figure 1). Our approach mines such substring pairs in input interaction data – which we termed the *correlated motifs *– that correspond to *x *and *y*. The term "correlated" indicates that the output motif pair may not necessarily be directly binding each other but their co-occurrences in interacting sequences are significant. Our new approach offers the following advantages:

1. In contrast to both OTM and MTM-based approaches, it simultaneously finds two motifs that are interaction-correlated instead of one motif.

2. Like the MTM approach, it increases the number of motif instances for detection (See Figure 1).

3. However, it does not require any prior knowledge for protein grouping (although, when available, such information would still be useful), resting on the assumption that members of a protein group should share similar substrings that can be extracted by our approach as one of the motifs (See Figure 1).

4. By finding pairs of correlated motifs in the interaction data instead of single motifs in protein sequence data, our approach is more stringent and hence more resilient against noise since it is less likely for two spurious noise-induced motifs to co-occur in the interaction data more frequently than the true ones.

We adopted the (*l*, *d*)-motif model which had been used frequently to model motifs in biological sequences thanks to its simplicity [17-22]. In the (*l*, *d*)-motif model, the actual motif and motif instances are strings of length *l *and each instance differs by no more than *d *mismatches from the actual motif. Thus any two motif instances would have at most 2*d *mismatches. Consequently, a set of very similar substrings can be modelled as a (*l*, *d*) motif with a small *d *while a more diverse substring set need to be modelled with a larger *d*. We then formulated our approach as an (*l*, *d*)-*motif pair finding *problem, and presented an exact algorithm, D-MOTIF, as well as its approximation algorithm, D-STAR to solve the problem.

**Figure 2:**
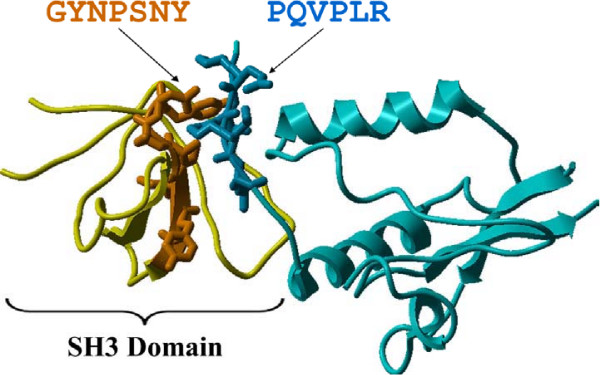
**Evidence from PDB structural data – SH3 domain vs. PxxPxR**. 3D structure (PDB ID: 1AVZ) of a SH3 domain of FYN tyrosine kinase bound to with another protein. The sequence segments that express the "PxxPxR" motif and "GxxPxNY" motif (detected by D-STAR in this work) are highlighted in dark blue and orange respectively. The two segments correspond to actual interacting subsequences.

**Figure 3:**
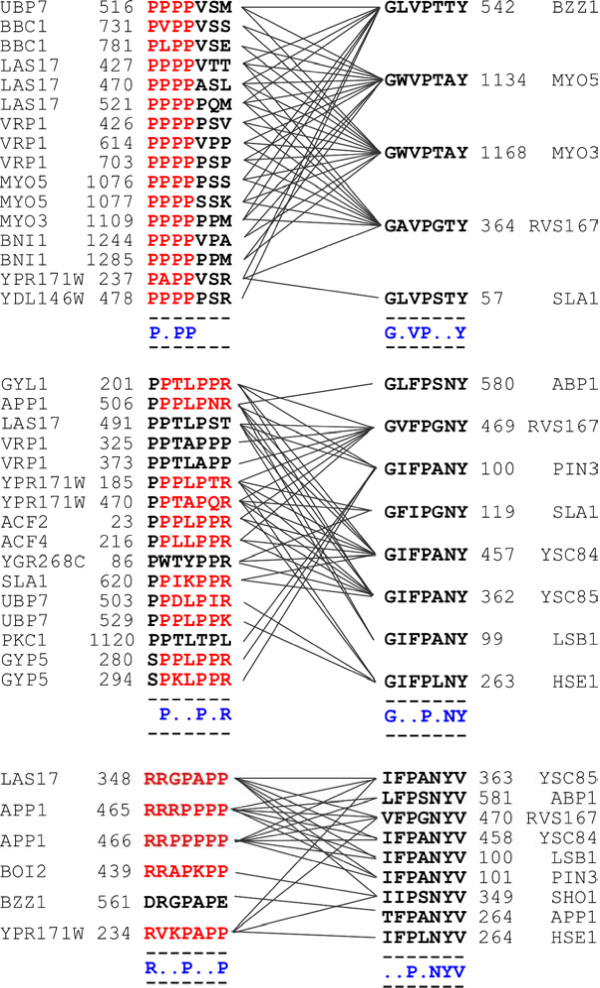
**The "PxxP", "PxxPx[KR]" and "[KR]xxPxxP" motifs and their associated motifs extracted by D-STAR**. Lines between the sequence segments denote interaction between their parent proteins. The result is found from multiple runs of D-STAR with different combination of motif width *l *= 6, 7, 8, distance *d *= 1 and *k*_*i *_= *k*_*n *_= 5. We then rank all the outputs from the different runs by their χ-score.

Our benchmarking analysis shows that D-STAR's performance is comparable to D-MOTIF's with a substantially shorter running time. Thus, in evaluation experiments, we compare only D-STAR with other existing algorithms so that we can run extensive tests on both simulated and real biological datasets. Result from the former validates that the correlated motif approach is more robust than OTM and MTM in extracting motifs from sparse but noisy interaction data. Evaluation on real biological datasets, on another hand, demonstrates that our D-STAR algorithm is able to extract correlated motifs that are biologically relevant. On a SH3 domain interaction dataset [3], D-STAR extracted "PxxPx[KR]" and "GxxPxNY" as correlated motifs; the two motifs were subsequently validated to actual interacting interfaces in the structural data of SH3 domain and its ligand (see Figure 2). D-STAR also extracted "[KR]xxPxxP", a known SH3 binding motif, that was not detected by any existing algorithms tested in this study(see Figure 3 and Table 1). Application of D-STAR on the TGF*β *signaling pathway [23] extracted correlated motifs that mapped to putative phosphorylation sites and kinase subregions in proteins respectively (more details in the Additional file 1).

**Table 1:** Comparison on the performance of various algorithms on the SH3 dataset

Algorithm	PxxP	PxxPx[KR]	[KR]xxPxxP
D-STAR	1^*st*^	1^*st*^	8^*th*^
S-STAR	1^*st*^	-	-
MEME	1^*st*^	-	-
GIBBS	3^*rd*^	3^*rd*^	-

Rank of sequence segment sets or sequence segment pair sets output by the various algorithms that express various known binding motifs of SH3 domains. "-" denote the biological motif is not expressed within the top 50 sequence segment sets.

### Related works

There are existing works [24-27] that also find over-represented pairs of co-occurring sequence patterns from protein-protein interaction data, but most focused on discovering interaction correlations between sequence patterns pre-defined in existing databases such as Pfam, InterPro and Prosite. Such usage of pre-defined patterns drastically reduces the motif search space to enable motif mining in large interaction network. However, their coverage is also consequently limited by the degree of completeness of existing pattern databases. To-date, only about 200 binding motifs out of some few thousands that possibly exist [2] have been found. The correlated motif approach outlined in this work can therefore complement existing works by discovering more novel motifs as well as their correlations from the increasingly abundant protein interaction data. Our algorithms can also be applied on biological pathways or protein networks directly to detect the most significant co-occurring motif pairs in these pathways. Such functionality is important for studying pathways known to be mediated by recurring domains and motifs, like various signaling pathways [28,29].

## Results and discussion

In the following discussion, we compared our algorithms (D-STAR and D-MOTIF) against the existing algorithms, run in either OTM or MTM mode. This is because, to our knowledge, there is no existing algorithm based on our approach. Recall that in the (*l*, *d*)-motif model, the motif (a consensus string) and its instances are strings of length *l *and each instance differs by no more than *d *mismatches from the actual motif. The *l *and *d *are two parameters to the algorithms. Users can either input specific *l *and *d *into the algorithms or input a range of values for *l *and *d *instead. In the latter, the algorithms will extract the different (*l*, *d*)-motif pairs and output them, ranked based on their significance. At the same time, user must provide two additional parameters *k*_*i *_and *k*_*n *_for more directed search: *k*_*i *_specifies the minimum number of interactions that (*l*, *d*)-motif pairs must co-occur in while *k*_*n *_dictate the minimum of interacting proteins that must express each of the (*l*, *d*) motif.

In short, our algorithms tries to cluster the interaction data into groups of interaction which express some statistically significant (*l*, *d*)-motif pair; it look for pairs of similar substring set (defined by the (*l*, *d*) motif model) occurring across pairs of interacting proteins, and rank them based on their co-occurrence statistical significance. The exact algorithm D-MOTIF would find all possible motif pairs which satisfy the threshold given while D-STAR would allow a bit of inaccuracy for the sake of speed. We performed a preliminary experiment on D-MOTIF and D-STAR to compare their accuracy and efficiency, and found out that D-MOTIF is only modestly more accurate than D-STAR while running several orders of magnitude slower than the latter. The details of the comparison can be found in the Methods section. For efficiency, we therefore only ran D-STAR in our following evaluation experiments.

### Artificial data with planted (*l*, *d*)-motifs

We evaluate the robustness of D-STAR against noise in input data using simulated data with planted (*l*, *d*)-motifs. Another goal of the study is to investigate the performance of D-STAR when dealing with problems involving weak motifs. This will provide insights to the user on how the latter influences D-STAR's accuracy.

#### Simulation setup

We follow the simulation setup devised in [17], where the authors planted well-defined artificial (*l*, *d*)-motifs into random sequences to create artificial datasets for evaluation. Here, we create sequences with planted (*l*, *d*)-motifs and then pair them up to generate artificial interaction datasets. For each pair of (*l*, *d*)-motifs (*x*, *y*), five instances of motif *x *and five instances of motif *y *are inserted into ten randomly selected protein sequences. To simulate the real scenarios as close as possible, the motifs were planted in randomly selected yeast (*Saccharomyces cerevisiae*) protein sequences instead of random sequences. Let us denote the five sequences with planted motif *x *as sequence set *P*_*x*_, and the five sequences with planted motif *y *as sequence set *P*_*y*_. We set |*P*_*x*_| = |*P*_*y*_| = 5 in our current simulations.

We simulate the real protein interactions by pairing every sequences in *P*_*x *_to N
 MathType@MTEF@5@5@+=feaafiart1ev1aaatCvAUfKttLearuWrP9MDH5MBPbIqV92AaeXatLxBI9gBamrtHrhAL1wy0L2yHvtyaeHbnfgDOvwBHrxAJfwnaebbnrfifHhDYfgasaacH8akY=wiFfYdH8Gipec8Eeeu0xXdbba9frFj0=OqFfea0dXdd9vqai=hGuQ8kuc9pgc9s8qqaq=dirpe0xb9q8qiLsFr0=vr0=vr0dc8meaabaqaciaacaGaaeqabaWaaeGaeaaakeaaimaacqWFneVtaaa@383B@ sequences in *P*_*y*_, and vice versa. A *spurious *interaction is modeled by pairing a protein in *P*_*x*_(*P*_*y*_, resp.) with a random yeast protein not in *P*_*y*_(*P*_*x*_, resp.). Given that a protein interacts with an average of 5.8 other proteins (interaction statistics in DIP [30]), and that the high throughput yeast two-hybrid technique is known to have at least 50% error [14], we would expect at most 2.9 true interactions per protein. Being conservative, we set N
 MathType@MTEF@5@5@+=feaafiart1ev1aaatCvAUfKttLearuWrP9MDH5MBPbIqV92AaeXatLxBI9gBamrtHrhAL1wy0L2yHvtyaeHbnfgDOvwBHrxAJfwnaebbnrfifHhDYfgasaacH8akY=wiFfYdH8Gipec8Eeeu0xXdbba9frFj0=OqFfea0dXdd9vqai=hGuQ8kuc9pgc9s8qqaq=dirpe0xb9q8qiLsFr0=vr0=vr0dc8meaabaqaciaacaGaaeqabaWaaeGaeaaakeaaimaacqWFneVtaaa@383B@ = 2 here. Let *ε *be the noise level defined as the fraction of the spurious interactions within all interactions that belong to one particular protein. We investigate the performance of the algorithms with *ε *= 0.50 as well as *ε *= 0.60. For instance, when N
 MathType@MTEF@5@5@+=feaafiart1ev1aaatCvAUfKttLearuWrP9MDH5MBPbIqV92AaeXatLxBI9gBamrtHrhAL1wy0L2yHvtyaeHbnfgDOvwBHrxAJfwnaebbnrfifHhDYfgasaacH8akY=wiFfYdH8Gipec8Eeeu0xXdbba9frFj0=OqFfea0dXdd9vqai=hGuQ8kuc9pgc9s8qqaq=dirpe0xb9q8qiLsFr0=vr0=vr0dc8meaabaqaciaacaGaaeqabaWaaeGaeaaakeaaimaacqWFneVtaaa@383B@ = 2 and *ε *= 0.50, the proteins in *P*_*x *_and *P*_*y *_will be involved in (on average) 4 interactions; two of which would be spurious.

#### The algorithms and parameter settings

We applied D-STAR, as well as other known motif extraction algorithms such as MEME and Gibbs Sampler to see whether they can extract instances of both planted motifs amongst its motif pairs with the highest scores from the noisy input datasets. We also implemented an algorithm, S-STAR, to find single (*l*, *d*)-motifs in subsets of protein sequences based on the well-established SP-STAR algorithm [17]. We ran MEME, Gibbs Sampler and S-STAR using the MTM approach since N
 MathType@MTEF@5@5@+=feaafiart1ev1aaatCvAUfKttLearuWrP9MDH5MBPbIqV92AaeXatLxBI9gBamrtHrhAL1wy0L2yHvtyaeHbnfgDOvwBHrxAJfwnaebbnrfifHhDYfgasaacH8akY=wiFfYdH8Gipec8Eeeu0xXdbba9frFj0=OqFfea0dXdd9vqai=hGuQ8kuc9pgc9s8qqaq=dirpe0xb9q8qiLsFr0=vr0=vr0dc8meaabaqaciaacaGaaeqabaWaaeGaeaaakeaaimaacqWFneVtaaa@383B@ = 2 is too low for an OTM-based approach to detect the motifs. We assume that all the algorithms using the MTM-approach will be ran only on the proteins that interact with those in *P*_*y *_when trying to find motif *x *(and vice versa for *y*). The average of the two cases is the reported performance. Note that this effectively provides the existing algorithms with prior knowledge on the underlying groupings of the protein sequences; the knowledge of sequence groups *P*_*x *_and *P*_*y*_.

To search for the set of planted (*l*, *d*)-motifs, we set the parameters for the various algorithms as follows. For MEME, the parameters are: *Mode = ZOOPS *(option in MEME when not every input sequences are guaranteed to contain a motif of interest) and *Motif Width *= *l*. For Gibb Sampler, the parameters are: *Mode = Motif Sampler *(option in Gibbs Sampler when not all input sequences are guaranteed to contain a motif of interest), *Motif Width *= *l *and *Expected Motif Occurrence *= 5. For D-STAR and S-STAR, being (*l*, *d*)-motif searching algorithms, the first two parameters are *l *and *d*. We set the minimum number of motif occurrences in the sequences, *k*_*n *_= 5. For D-STAR, the minimum number of interactions between the instances of the correlated motifs, *k*_*i *_is also set to 5 as well.

#### Evaluation metrics

We evaluate the relative performance of the algorithms using the following metrics:

Specificity=TPx+TPyTPx+TPy+FPx+FPySensitivity=TPx+TPyTPx+TPy+FNx+FNyF-Measure=(2×Specificity×Sensitivity)(Specificity+Sensitivity)
 MathType@MTEF@5@5@+=feaafiart1ev1aaatCvAUfKttLearuWrP9MDH5MBPbIqV92AaeXatLxBI9gBaebbnrfifHhDYfgasaacH8akY=wiFfYdH8Gipec8Eeeu0xXdbba9frFj0=OqFfea0dXdd9vqai=hGuQ8kuc9pgc9s8qqaq=dirpe0xb9q8qiLsFr0=vr0=vr0dc8meaabaqaciaacaGaaeqabaqabeGadaaakeaafaqaaeWabaaabaGaee4uamLaeeiCaaNaeeyzauMaee4yamMaeeyAaKMaeeOzayMaeeyAaKMaee4yamMaeeyAaKMaeeiDaqNaeeyEaKNaeyypa0ZaaSaaaeaacqWGubavcqWGqbaudaWgaaWcbaGaemiEaGhabeaakiabgUcaRiabdsfaujabdcfaqnaaBaaaleaacqWG5bqEaeqaaaGcbaGaemivaqLaemiuaa1aaSbaaSqaaiabdIha4bqabaGccqGHRaWkcqWGubavcqWGqbaudaWgaaWcbaGaemyEaKhabeaakiabgUcaRiabdAeagjabdcfaqnaaBaaaleaacqWG4baEaeqaaOGaey4kaSIaemOrayKaemiuaa1aaSbaaSqaaiabdMha5bqabaaaaaGcbaGaee4uamLaeeyzauMaeeOBa4Maee4CamNaeeyAaKMaeeiDaqNaeeyAaKMaeeODayNaeeyAaKMaeeiDaqNaeeyEaKNaeyypa0ZaaSaaaeaacqWGubavcqWGqbaudaWgaaWcbaGaemiEaGhabeaakiabgUcaRiabdsfaujabdcfaqnaaBaaaleaacqWG5bqEaeqaaaGcbaGaemivaqLaemiuaa1aaSbaaSqaaiabdIha4bqabaGccqGHRaWkcqWGubavcqWGqbaudaWgaaWcbaGaemyEaKhabeaakiabgUcaRiabdAeagjabd6eaonaaBaaaleaacqWG4baEaeqaaOGaey4kaSIaemOrayKaemOta40aaSbaaSqaaiabdMha5bqabaaaaaGcbaGaeeOrayKaeeyla0Iaeeyta0KaeeyzauMaeeyyaeMaee4CamNaeeyDauNaeeOCaiNaeeyzauMaeyypa0ZaaSaaaeaacqGGOaakcqaIYaGmcqGHxdaTcqqGtbWucqqGWbaCcqqGLbqzcqqGJbWycqqGPbqAcqqGMbGzcqqGPbqAcqqGJbWycqqGPbqAcqqG0baDcqqG5bqEcqGHxdaTcqqGtbWucqqGLbqzcqqGUbGBcqqGZbWCcqqGPbqAcqqG0baDcqqGPbqAcqqG2bGDcqqGPbqAcqqG0baDcqqG5bqEcqGGPaqkaeaacqGGOaakcqqGtbWucqqGWbaCcqqGLbqzcqqGJbWycqqGPbqAcqqGMbGzcqqGPbqAcqqGJbWycqqGPbqAcqqG0baDcqqG5bqEcqGHRaWkcqqGtbWucqqGLbqzcqqGUbGBcqqGZbWCcqqGPbqAcqqG0baDcqqGPbqAcqqG2bGDcqqGPbqAcqqG0baDcqqG5bqEcqGGPaqkaaaaaaaa@D573@

where *TP*_*x*_(*TP*_*y*_, resp.) is the number of correctly recovered planted motifs *x*(*y*, resp.) *FN*_*x*_(*FN*_*y*_, resp.) is the number of instances of the planted motif *x*(*y*, resp.) the algorithm fails to recover. Lastly, *FP*_*x*_(*FP*_*y*_, resp.) is the number of spurious motifs included by the algorithm as a candidate instance of *x*(*y*, resp.).

#### Results

We applied D-STAR and all the other algorithms on numerous sets of simulated interaction data with different planted (*l*, *d*)-motifs, namely the (8, 1), (7, 1), (9, 2), (6, 1) and (8, 2)-motifs (listed in decreasing order of motif strength). For each combination of motif and *ε *value, we generated 10 random datasets and compute the average performance of the algorithms in discovering correct motif. Our results showed that MEME and Gibbs Sampler performed quite poorly. Even for a relatively strong (8, 1)-motif, MEME can only achieve F-Measures of 0.49 and 0.35 for *ε *= 0.50 and 0.60, respectively (As for Gibbs Sampler, the F-Measures were 0.58 and 0.29 respectively). However, since both of these algorithms used different motif models, they may not be optimized to search for (*l*, *d*)-motifs. Instead, we will compare their relative performance on real biological data later. An noteworthy observation, however, is increased noise in input data can drastically decrease the performances of the algorithms.

**Figure 4:**
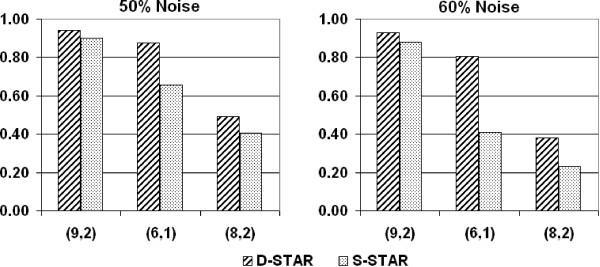
**Comparison between D-STAR and S-STAR**. Comparison between D-STAR and S-STAR(A variant of SP-STAR) in extracting planted (*l*, *d*)-motifs. The motifs are arranged on the x-axis in decreasing order of motif strength. The number of planted motif instances in each dataset is 5 and the datapoint is the average over 10 runs.

Not surprisingly, both D-STAR and S-STAR attained very high average F-Measure of 0.99 for relatively stronger (8, 1) and (7, 1) – motifs on all values of *ε *(data not shown). Figure 4 shows the comparison of F-Measures of D-STAR and S-STAR on the weaker (9, 2), (6, 1) and (8, 2) motifs. Observe that D-STAR performed consistently better than S-STAR on all the cases, and furthermore, the performance margins were higher when there were more noise in the data. This study validates that even without having the prior knowledge of the motifs contained in the interaction data, D-STAR is able to handle noise much better than the other algorithms. This is of practical importance since real interaction data are often highly noisy data containing many interactions between unknown domains and/or motifs.

### Biological data

In this section, we apply our algorithm on two biologically significant datasets: SH3 domain interaction data and TGF*β *signaling pathway data. We show that our approach can better discover real biological motifs than the other methods.

#### SH3 domain interaction data

SH3 domains are conserved amino acid segments (of length ≈ 60 amino acids) found across multiple proteins. Through various biological experiments, SH3 domains have been determined to bind short sequence segments expressing the general motif "PxxP" [3]. The interactions between SH3 proteins and the "PxxP" motif mirror our motif pair (*x*, *y*) (in this case, one of the motifs should correspond to parts of SH3 domain). For evaluation, we use the same dataset derived by Tong *et. al. *to find the interacting partners of SH3 domain proteins [3]. This dataset, which we called SH3-PxxP-Tong, was downloaded from BIND online database. It consists of 233 protein-protein interactions among 146 yeast proteins of which 23 are SH3 domain proteins (as determined using HMMER program from Pfam). We will first assess whether the known SH3 binding motifs can be extracted among the top motifs by each algorithm. Next, we investigate the biological relevance of the correlated motifs extracted by D-STAR.

#### The algorithms and parameters

We ran D-STAR on the SH3-PxxP-Tong dataset multiple times with different combinations of *l *= 6, 7, 8, *d *= 1 and *k*_*n *_= *k*_*i *_= 5. The outputs from the different runs were then systematically ranked using their χ-scores. Note again that in the case of our D-STAR algorithm, the motifs were mined without having to separate the SH3 domain proteins and the non-SH3 domain proteins, unlike the other MTM motif extraction methods which require such prior knowledge. For comparison, we also attempted to extract the "PxxP"-like motifs with MEME (*ZOOPS mode, Motif Width *= 4 – 9), Gibbs Sampler (*Motif Sampler mode, Motif Width *= 4 – 8, *Expected Motif Number *≥ 5) and SP-STAR (*l *= 6, 7, 8, *d *= 1 and *Minimum Motif Number *= 5) from the 130 sequences in the dataset that bind to any SH3 proteins (*the MTM approach*).

#### Validation

Without the luxury of experimentally validating the motifs extracted, it is hard to determine the accuracy of the various algorithms correctly. However, we reasoned that a good algorithm should at least extract most of the known motifs. In other words, when applying D-STAR on the interaction data of SH3 proteins, we should expect it to extract some "PxxP"-like motifs on one side and another motif that occurs consistently in SH3 domains on the other side. We consider here the well-known SH3-binding motifs "PxxP", "PxxPx[RK]" and "[RK]xxPxxP". For each of these three motifs, we check whether it was "expressed" within the top 50 motifs reported (usually user would not want to check beyond this number). We define a set of protein sequence segments reported by an algorithm to be expressing a motif if at least 50% of the sequence segments match the pattern.

#### Results

Table 1 shows the results for D-STAR, S-STAR, MEME, and Gibbs Sampler. The generic "PxxP" motif was extracted among the top outputs by all algorithms. However, only our D-STAR algorithm managed to extract both "PxxPx[KR]" and "[KR]xxPxxP" motifs (within the top 50 motifs output of each algorithm). In fact, only two instances of the "PxxPx[KR]" motif are found in the segments extracted within the top 50 sets of segments extracted by MEME. No "[KR]xxPxxP" motif instance was extracted. To be sure, we re-ran MEME on the same 130 sequences with more specific motif lengths = 6–7 (instead of motif length = 4–9) but to no avail. This confirmed that MEME with the MTM approach has indeed missed out the more specific variants. As for S-STAR, the limited instances of the "PxxPx[KR]" and "[KR]xxPxxP" motifs extracted were overwhelmed by the more general "PxxP" motif. D-STAR, despite having no access to prior grouping knowledge unlike the other algorithms, was the only algorithm that was able to extract the specific SH3-binding motifs.

One might argue that since the MTM-algorithms were applied on the set of all SH3-binding sequences which contained either of the motifs "PxxPx[KR]" and "[KR]xxPxxP", it may be unsurprising that only the general "PxxP" motif was extracted instead of the more specific motifs. The OTM approach may be more suitable for extracting the specific motifs since it does not consider the SH3-binding sequences in a "wholesale" manner as the MTM approach. As such, we applied MEME, Gibbs Sampler and S-STAR on the interacting protein partners of each individual SH3 protein in the SH3-PxxP dataset. In total, the OTM approach can be applied on the 22 SH3 proteins that bind more than 1 protein sequence. We used the same parameters used in the MTM approach for each algorithm except that the *Minimum Motif Occurrence *= 2. We deemed a motif to be extracted successfully if more than 50% of a segment set within the top 50 sets extracted expressed the motif and that 50% should comprise of at least 2 instances. For MEME, "PxxP" motif was extracted for 3 SH3 proteins (Abp1,Rvs167,Bzz1) and "PxxPx[KR]" motif was extracted for 2 other SH3 proteins (Ysc84,Myo3). Gibbs Sampler extracted the "PxxP" and "PxxPx[KR]" motifs for 1(Sho1) and 2 SH3 proteins (Yfr024c,Ysc84) respectively. Finally, for S-STAR, the "PxxP" motif was extracted for 8 SH3 proteins (Fus1,Bbc1,Rvs167,Hse1,Bzz1,Myo3,Hof1,Nyo5) and the "PxxPx[KR]" motif was extracted for 2 other SH3 proteins (Yfr024c,Ysc84). Again, all the algorithms failed to extract "[KR]xxPxxP" motif within the top 50 output for any of the SH3 proteins. In comparison, D-STAR extracted the specific "PxxPx[KR]" and "[KR]xxPxxP" for more SH3 proteins (Figure 3).

Since D-STAR extracts correlated motifs, it is interesting to further analyze the extracted associated sequence segments of the three proline-rich motifs as shown in Figure 3. We were intrigued to discovered that all associated sequence segments extracted together with "PxxP", "PxxPx[RK]" and "[RK]xxPxxP" by D-STAR were found within SH3 domains. In addition, we also discovered that all associated sequence segments of the three proline-rich motifs expressed a "PxxY" general consensus. Specifically, D-STAR extracted "GxxPxNY" as the associated motif of "PxxPx[KR]" motif. A further check into the structural data (PDB ID:1AVZ) of an experimentally determined interaction between an SH3 protein and a protein expressing a "PxxPx[KR]" motif reveals that the sequence segment in SH3 domain expressing the "GxxPxNY" motif indeed forms a binding interface with the segment expressing the "PxxPx[RK]" motif (Figure 2). Hence, in this particular case, D-STAR has extracted correlated motifs that actually are binding motifs.

#### TGF*β *signaling pathway

Next, we applied D-STAR on the interaction network of TGF*β *signaling pathway that was derived using LUMIER [23] – an automated high-throughput protein interaction detection technology that can detect phosphorylation-dependent interactions. Note that the original experiment was not specifically geared toward detecting interactions of any particular protein domain or motif. Hence, unlike the SH3-PxxP dataset, it is not immediately apparent whether any relevant motif pairs can be found in the interaction network. We applied D-STAR on this interaction dataset to see whether we can extract any interesting motif pairs. The dataset was retrieved from BIND database and consists of 446 interactions among 214 proteins. D-STAR was applied on the dataset with the same parameters used for SH3-PxxP dataset. As we do not know what to expect as correct answer, we focused on validating the top motif pair extracted. Interestingly, D-STAR extracted a motif pair, with general consensus patterns "[TA]E[LI]Y[NQ]T" and "GKT[CIS][ILT][IL]", from 87 unique interactions as our top output (Additional file 1). For ease of discussion, let us denote the motif pair as (*X*, *Y*). First, we verified that (*X*, *Y*) is not likely to occur by chance as the estimated probability (p-value) of getting the motif pair with the same interaction set size is less than 0.001 (by testing the motif pair on 1000 randomly generated interaction data with the same network topology and sequences). Hence, we conjectured that the motif pair is a possible key interaction mechanism in the TGF*β *signaling pathway.

We also found that the sequence segment set of motif *Y *is enriched in known kinase phosphorylation motifs (27 sites in 50 segments, based on result from PhosphoMotif Finder [31]). To determine the significance of finding 27 sites in the segment sets, we generate 1000 segments sets, each containing 50 segments randomly selected from the same protein set. We found out that none of them contain at least 27 segments with the phosphorylation motifs, implying an estimated *p*-value < 0.001.

We listed the over-represented phosphorylation motifs in Table 2 (for a detailed listings of all of the phosphorylation sites, see Additional file 1). Further analysis also showed that 5 out of 6 associated sequence segments of motif *X *were also found within kinase protein domains (determined using HMMER from Pfam). Such biological characterization of our extracted motif pair (*X*, *Y*) with *X *as kinase motifs and *Y *as phosphorylation motifs is indeed in concurrence with the fact that signalling pathways are typically regulated by kinases through protein phosphorylation. This further indicates that our method have extracted a biologically feasible motif pair from the TGF*β *interaction dataset.

**Table 2:** The over-represented phosphorylation sites motifs found by D-STAR

Motif	Expected	Observed	Odd-Ratio
[R/K]x[S/T]	3.15	17	5.40
Kxx[S/T]	1.22	6	4.92

The motifs of the phosphorylation sites that are over-represented in the segment set with the general pattern "GKT[CIS][ILT][IL]". As the motifs are degenerate, we compared their actual number of occurrence with their expected random occurrence within any random segment set of the same size preserving the same amino acid distribution as the whole dataset's.

We also investigated whether such kinase phosphorylation motifs may also be extracted using the OTM approach. For each kinase protein found in *Y *by D-STAR, we submitted their binding partners to MEME (*ZOOPS mode, Motif Width *= 4 – 8), Gibbs Sampler (*Motif Sampler mode, Motif Width *= 4 – 8, *Expected Motif Number *≥ 2) and S-STAR (*l *= 6, 7, 8, *d *= 1 and *k*_*n *_= 5). We found that over-represented phosphorylation motifs can be found within the top 10 output segment sets for only 2 out of the 5 kinase proteins by all MEME, Gibbs Sampler and S-STAR (based on result from PhosphoMotif Finder).

Note that the above OTM approach had relied on the pre-grouping of kinase proteins to guide the motif discovery (and yet its result were still not as good as our D-STAR's motifs). In practice, such specific prior biological knowledge may not be available. In this case, in order to discover that (*X*, *Y*) is a significant interaction mechanism in the TGF*β *signaling pathway, one would first need to repeatedly mine motifs in all possible groupings of the protein sequences before finding some significant correlations between the motifs extracted from the protein groups. This can be a laborious process – even if we were to use the proteins' domain information for pre-grouping the proteins, there could be a large number of domains involved, while the performance may be limited by the coverage of domain information. D-STAR, on the other hand, depends on no such information and found the correlated motif pairs directly from input interaction data in one single process.

## Conclusion

Discovery of novel binding motifs acting as interaction switches for biological circuits can lead to invaluable insights for important applications such as drug discovery, as various short binding motifs have been found to be associated with disease pathways. However, such motifs have also been known to be hard to find both experimentally and computationally [2].

The recently available protein-protein interaction data present a rich data source to aid in such important discoveries through motif discovery algorithms. The efforts can be hindered by sparse and noisy nature of existing protein interaction data, as well as the inadequacy of current biological knowledge. In this paper, we have proposed a novel approach of mining correlated *de – novo *motifs from interaction data. We formulated our approach as an (*l*, *d*)-motif pair finding problem for which we gave an exact algorithm, D-MOTIF, as well as its approximation algorithm, D-STAR. Our evaluation results have shown that our proposed approach can eliminates the need for prior knowledge on protein groups during the discovery process. Such functionality allows the discovery of motifs not to be constrained by inadequate biological knowledge. The approach is also more robust in extracting motifs from noisy interaction data. Of course, since D-STAR is devised for finding linear sequence motifs, it would fail if one of the correlated motifs is a structural one. However, it may still be used to identify short conserved sequence regions that formed parts of such structural motifs. Given that existing protein structural data is still very limited when compared to available protein-protein interaction data, short conserved sequence regions identified by D-STAR could facilitate further biological experiments like mutagenesis studies.

While we have presented an approximation algorithm D-STAR to speed up the extraction of motif pairs from interaction data, more work will need to be done in order to scale up the approach to handle genome-wide interaction data or the larger DNA-protein interaction data. Also, as real biological motifs can be of varying lengths, we will also need to extend our current approach to discover binding motifs that are not of any predefined lengths. We leave these as future work.

## Methods

### Preliminaries

Let *s *= *a*_1_*a*_2_*a*_3_...*a*_*n *_be a length-*n *protein sequence defined over the alphabet Σ of 20 amino acids, and *s*[*u*, *v*] as the substring of the string *s *starting at position *u *up to position *v*. When the substring's length *l *is fixed, we simply write *s*[*u*] for *s*[*u*, *u *+ *l *- 1]. We will call such a substring the *l*-substring at position *u*.

#### The (*l*, *d*)-motif finding problem

The definition of (*l*, *d*)-motif was originally proposed in [17] to model motifs in biological sequences. Consider a set *S *= {*s*_1_, *s*_2_, *s*_3_...,*s_*t*_*} of *t *protein sequences of length *n*. A length-*l *pattern *p *is an (*l*, *d*)-motif in *S' *⊆ *S *if all sequences *s*_*i *_∈ *S' *have at least one *l*-substring *s*_*i*_[*u*] which differs from *p *by at most *d *mismatches. Such *s*_*i*_[*u*]'s are termed as the *instances *of *p*. In their work, Pevzner *et. al. *[17] computed for the (*l*, *d*)-motif *p *that has at least one instance in each sequence in S. In our work, it is important to find motifs from a significantly large subset *S' *of *S *since, in some case, there is no guarantee that every input sequence would contain an instance of the motif. In other words, for a given (*l*, *d*)-motif *p*, let S
 MathType@MTEF@5@5@+=feaafiart1ev1aaatCvAUfKttLearuWrP9MDH5MBPbIqV92AaeXatLxBI9gBamrtHrhAL1wy0L2yHvtyaeHbnfgDOvwBHrxAJfwnaebbnrfifHhDYfgasaacH8akY=wiFfYdH8Gipec8Eeeu0xXdbba9frFj0=OqFfea0dXdd9vqai=hGuQ8kuc9pgc9s8qqaq=dirpe0xb9q8qiLsFr0=vr0=vr0dc8meaabaqaciaacaGaaeqabaWaaeGaeaaakeaaimaacqWFse=uaaa@3845@_*d*_(*p*) be {*s *∈ *S *| *s *contains an *l*-substring of distance at most *d *from *p*}. Given the minimum number of instance threshold *k*_*n*_, we then define the general (*l*, *d*)-motif finding problem as finding all (*l*, *d*)-motif *p *in *S *such that |S
 MathType@MTEF@5@5@+=feaafiart1ev1aaatCvAUfKttLearuWrP9MDH5MBPbIqV92AaeXatLxBI9gBamrtHrhAL1wy0L2yHvtyaeHbnfgDOvwBHrxAJfwnaebbnrfifHhDYfgasaacH8akY=wiFfYdH8Gipec8Eeeu0xXdbba9frFj0=OqFfea0dXdd9vqai=hGuQ8kuc9pgc9s8qqaq=dirpe0xb9q8qiLsFr0=vr0=vr0dc8meaabaqaciaacaGaaeqabaWaaeGaeaaakeaaimaacqWFse=uaaa@3845@_*d*_(*p*)| ≥ *k*_*n*_.

#### The (*l*, *d*)-motif pair finding problem

We extend the problem of finding (*l*, *d*)-motifs in a set of sequences into one for finding motif *pairs *in a set of sequence *pairs *for mining interacting motifs in a set of protein-protein interactions. Given a protein interaction dataset *I *⊆ *S *× *S *of size *m *over the set of proteins *S *where for any (*s*_*i*_, *s*_*j*_) ∈ *I *we have *i *≤ *j*, we want to find a pair of (*l*, *d*)-motifs which is over-represented in *I*. That is, we want to find an (*l*, *d*)-motif pair (*x*, *y*) that have the following characteristics:

(1) Let *I*_(*x*, *y*) _be the set of interactions between S
 MathType@MTEF@5@5@+=feaafiart1ev1aaatCvAUfKttLearuWrP9MDH5MBPbIqV92AaeXatLxBI9gBamrtHrhAL1wy0L2yHvtyaeHbnfgDOvwBHrxAJfwnaebbnrfifHhDYfgasaacH8akY=wiFfYdH8Gipec8Eeeu0xXdbba9frFj0=OqFfea0dXdd9vqai=hGuQ8kuc9pgc9s8qqaq=dirpe0xb9q8qiLsFr0=vr0=vr0dc8meaabaqaciaacaGaaeqabaWaaeGaeaaakeaaimaacqWFse=uaaa@3845@_*d*_(*x*) and S
 MathType@MTEF@5@5@+=feaafiart1ev1aaatCvAUfKttLearuWrP9MDH5MBPbIqV92AaeXatLxBI9gBamrtHrhAL1wy0L2yHvtyaeHbnfgDOvwBHrxAJfwnaebbnrfifHhDYfgasaacH8akY=wiFfYdH8Gipec8Eeeu0xXdbba9frFj0=OqFfea0dXdd9vqai=hGuQ8kuc9pgc9s8qqaq=dirpe0xb9q8qiLsFr0=vr0=vr0dc8meaabaqaciaacaGaaeqabaWaaeGaeaaakeaaimaacqWFse=uaaa@3845@_*d*_(*y*), namely, *I*_(*x*, *y*) _= *I *∩ (S
 MathType@MTEF@5@5@+=feaafiart1ev1aaatCvAUfKttLearuWrP9MDH5MBPbIqV92AaeXatLxBI9gBamrtHrhAL1wy0L2yHvtyaeHbnfgDOvwBHrxAJfwnaebbnrfifHhDYfgasaacH8akY=wiFfYdH8Gipec8Eeeu0xXdbba9frFj0=OqFfea0dXdd9vqai=hGuQ8kuc9pgc9s8qqaq=dirpe0xb9q8qiLsFr0=vr0=vr0dc8meaabaqaciaacaGaaeqabaWaaeGaeaaakeaaimaacqWFse=uaaa@3845@_*d*_(*x*) × S
 MathType@MTEF@5@5@+=feaafiart1ev1aaatCvAUfKttLearuWrP9MDH5MBPbIqV92AaeXatLxBI9gBamrtHrhAL1wy0L2yHvtyaeHbnfgDOvwBHrxAJfwnaebbnrfifHhDYfgasaacH8akY=wiFfYdH8Gipec8Eeeu0xXdbba9frFj0=OqFfea0dXdd9vqai=hGuQ8kuc9pgc9s8qqaq=dirpe0xb9q8qiLsFr0=vr0=vr0dc8meaabaqaciaacaGaaeqabaWaaeGaeaaakeaaimaacqWFse=uaaa@3845@_*d*_(*y*)). We require that |*I*_(*x*, *y*)_| ≥ *k*_*i *_for a minimum number of interaction threshold *k*_*i*_.

(2) Let S′d
 MathType@MTEF@5@5@+=feaafiart1ev1aaatCvAUfKttLearuWrP9MDH5MBPbIqV92AaeXatLxBI9gBamrtHrhAL1wy0L2yHvtyaeHbnfgDOvwBHrxAJfwnaebbnrfifHhDYfgasaacH8akY=wiFfYdH8Gipec8Eeeu0xXdbba9frFj0=OqFfea0dXdd9vqai=hGuQ8kuc9pgc9s8qqaq=dirpe0xb9q8qiLsFr0=vr0=vr0dc8meaabaqaciaacaGaaeqabaWaaeGaeaaakeaaimaacuWFse=ugaqbamaaBaaaleaacqWGKbazaeqaaaaa@39CE@(*x*) be a subset of S
 MathType@MTEF@5@5@+=feaafiart1ev1aaatCvAUfKttLearuWrP9MDH5MBPbIqV92AaeXatLxBI9gBamrtHrhAL1wy0L2yHvtyaeHbnfgDOvwBHrxAJfwnaebbnrfifHhDYfgasaacH8akY=wiFfYdH8Gipec8Eeeu0xXdbba9frFj0=OqFfea0dXdd9vqai=hGuQ8kuc9pgc9s8qqaq=dirpe0xb9q8qiLsFr0=vr0=vr0dc8meaabaqaciaacaGaaeqabaWaaeGaeaaakeaaimaacqWFse=uaaa@3845@_*d*_(*x*) containing sequences that interact with those in S
 MathType@MTEF@5@5@+=feaafiart1ev1aaatCvAUfKttLearuWrP9MDH5MBPbIqV92AaeXatLxBI9gBamrtHrhAL1wy0L2yHvtyaeHbnfgDOvwBHrxAJfwnaebbnrfifHhDYfgasaacH8akY=wiFfYdH8Gipec8Eeeu0xXdbba9frFj0=OqFfea0dXdd9vqai=hGuQ8kuc9pgc9s8qqaq=dirpe0xb9q8qiLsFr0=vr0=vr0dc8meaabaqaciaacaGaaeqabaWaaeGaeaaakeaaimaacqWFse=uaaa@3845@_*d*_(*y*). Similarly, let S′d
 MathType@MTEF@5@5@+=feaafiart1ev1aaatCvAUfKttLearuWrP9MDH5MBPbIqV92AaeXatLxBI9gBamrtHrhAL1wy0L2yHvtyaeHbnfgDOvwBHrxAJfwnaebbnrfifHhDYfgasaacH8akY=wiFfYdH8Gipec8Eeeu0xXdbba9frFj0=OqFfea0dXdd9vqai=hGuQ8kuc9pgc9s8qqaq=dirpe0xb9q8qiLsFr0=vr0=vr0dc8meaabaqaciaacaGaaeqabaWaaeGaeaaakeaaimaacuWFse=ugaqbamaaBaaaleaacqWGKbazaeqaaaaa@39CE@(*y*) be a subset of S
 MathType@MTEF@5@5@+=feaafiart1ev1aaatCvAUfKttLearuWrP9MDH5MBPbIqV92AaeXatLxBI9gBamrtHrhAL1wy0L2yHvtyaeHbnfgDOvwBHrxAJfwnaebbnrfifHhDYfgasaacH8akY=wiFfYdH8Gipec8Eeeu0xXdbba9frFj0=OqFfea0dXdd9vqai=hGuQ8kuc9pgc9s8qqaq=dirpe0xb9q8qiLsFr0=vr0=vr0dc8meaabaqaciaacaGaaeqabaWaaeGaeaaakeaaimaacqWFse=uaaa@3845@_*d*_(*y*) with interacting sequences with S
 MathType@MTEF@5@5@+=feaafiart1ev1aaatCvAUfKttLearuWrP9MDH5MBPbIqV92AaeXatLxBI9gBamrtHrhAL1wy0L2yHvtyaeHbnfgDOvwBHrxAJfwnaebbnrfifHhDYfgasaacH8akY=wiFfYdH8Gipec8Eeeu0xXdbba9frFj0=OqFfea0dXdd9vqai=hGuQ8kuc9pgc9s8qqaq=dirpe0xb9q8qiLsFr0=vr0=vr0dc8meaabaqaciaacaGaaeqabaWaaeGaeaaakeaaimaacqWFse=uaaa@3845@_*d*_(*x*). We also require that |S′d
 MathType@MTEF@5@5@+=feaafiart1ev1aaatCvAUfKttLearuWrP9MDH5MBPbIqV92AaeXatLxBI9gBamrtHrhAL1wy0L2yHvtyaeHbnfgDOvwBHrxAJfwnaebbnrfifHhDYfgasaacH8akY=wiFfYdH8Gipec8Eeeu0xXdbba9frFj0=OqFfea0dXdd9vqai=hGuQ8kuc9pgc9s8qqaq=dirpe0xb9q8qiLsFr0=vr0=vr0dc8meaabaqaciaacaGaaeqabaWaaeGaeaaakeaaimaacuWFse=ugaqbamaaBaaaleaacqWGKbazaeqaaaaa@39CE@(*x*)|, |S′d
 MathType@MTEF@5@5@+=feaafiart1ev1aaatCvAUfKttLearuWrP9MDH5MBPbIqV92AaeXatLxBI9gBamrtHrhAL1wy0L2yHvtyaeHbnfgDOvwBHrxAJfwnaebbnrfifHhDYfgasaacH8akY=wiFfYdH8Gipec8Eeeu0xXdbba9frFj0=OqFfea0dXdd9vqai=hGuQ8kuc9pgc9s8qqaq=dirpe0xb9q8qiLsFr0=vr0=vr0dc8meaabaqaciaacaGaaeqabaWaaeGaeaaakeaaimaacuWFse=ugaqbamaaBaaaleaacqWGKbazaeqaaaaa@39CE@(*y*)| ≥ *k*_*n*_.

We call this problem the (*l*, *d*)*-motif pair finding problem*. For every (*s*_*i*_, *s*_*j*_) ∈ *I*_(*x*, *y*)_, we want find (*s*_*i*_[*u*], *s*_*j*_[*v*]) which are instances of *x *and *y*. Biologically, (*s*_*i*_[*u*], *s*_*j*_[*v*]) may correspond to the functional regions in the proteins *s*_*i *_and *s*_*j *_that mediate their interaction.

#### Scoring function

It is likely for many (*l*, *d*)-motif pairs (*x*, *y*) to exist within a given interaction dataset *I *over the set of proteins *S*. We define here a scoring function to rank them systematically.

Let *O*(S_*x*_, S_*y*_) be the observed number of interactions between two protein sets S_*x *_and S_*y *_containing the motifs *x *and *y *respectively. Let *E*(S_*x*_, S_*y*_) be the expected number of interactions between S_*x *_and S_*y*_. We estimate *E*(S_*x*_, S_*y*_) based on the assumption that interactions occur at random. Since the probability of any interaction occurring between two random proteins in *S *is |I|(|S|2)
 MathType@MTEF@5@5@+=feaafiart1ev1aaatCvAUfKttLearuWrP9MDH5MBPbIqV92AaeXatLxBI9gBaebbnrfifHhDYfgasaacH8akY=wiFfYdH8Gipec8Eeeu0xXdbba9frFj0=OqFfea0dXdd9vqai=hGuQ8kuc9pgc9s8qqaq=dirpe0xb9q8qiLsFr0=vr0=vr0dc8meaabaqaciaacaGaaeqabaqabeGadaaakeaadaWcaaqaaiabcYha8jabdMeajjabcYha8bqaamaabmaabaqbaeqabiqaaaqaaiabcYha8jabdofatjabcYha8bqaaiabikdaYaaaaiaawIcacaGLPaaaaaaaaa@378E@, we have

E(Sx,Sy)=|I|(|S|2)[|Sx||Sy|−(|Sx∩Sy|2)]
 MathType@MTEF@5@5@+=feaafiart1ev1aaatCvAUfKttLearuWrP9MDH5MBPbIqV92AaeXatLxBI9gBaebbnrfifHhDYfgasaacH8akY=wiFfYdH8Gipec8Eeeu0xXdbba9frFj0=OqFfea0dXdd9vqai=hGuQ8kuc9pgc9s8qqaq=dirpe0xb9q8qiLsFr0=vr0=vr0dc8meaabaqaciaacaGaaeqabaqabeGadaaakeaacqWGfbqrcqGGOaakcqqGtbWudaWgaaWcbaGaemiEaGhabeaakiabcYcaSiabbofatnaaBaaaleaacqWG5bqEaeqaaOGaeiykaKIaeyypa0ZaaSaaaeaacqGG8baFcqWGjbqscqGG8baFaeaadaqadaqaauaabeqaceaaaeaacqGG8baFcqWGtbWucqGG8baFaeaacqaIYaGmaaaacaGLOaGaayzkaaaaamaadmaabaGaeiiFaWNaee4uam1aaSbaaSqaaiabdIha4bqabaGccqGG8baFcqGG8baFcqqGtbWudaWgaaWcbaGaemyEaKhabeaakiabcYha8jabgkHiTmaabmaabaqbaeqabiqaaaqaaiabcYha8jabbofatnaaBaaaleaacqWG4baEaeqaaOGaeyykICSaee4uam1aaSbaaSqaaiabdMha5bqabaGccqGG8baFaeaacqaIYaGmaaaacaGLOaGaayzkaaaacaGLBbGaayzxaaaaaa@5D6C@

where the term in the brackets computes the total number of interactions possible between the proteins in S_*x *_and S_*y*_. Based on the idea of χ^2^-statistic, we formulate the following function χ to score a given pair of (*x*, *y*)-motif containing protein sets S_*x *_and S_*y *_as

χ(Sx,Sy)=[O(Sx,Sy)−E(Sx,Sy)]2E(Sx,Sy)
 MathType@MTEF@5@5@+=feaafiart1ev1aaatCvAUfKttLearuWrP9MDH5MBPbIqV92AaeXatLxBI9gBaebbnrfifHhDYfgasaacH8akY=wiFfYdH8Gipec8Eeeu0xXdbba9frFj0=OqFfea0dXdd9vqai=hGuQ8kuc9pgc9s8qqaq=dirpe0xb9q8qiLsFr0=vr0=vr0dc8meaabaqaciaacaGaaeqabaqabeGadaaakeaacqaHhpWycqGGOaakcqqGtbWudaWgaaWcbaGaemiEaGhabeaakiabcYcaSiabbofatnaaBaaaleaacqWG5bqEaeqaaOGaeiykaKIaeyypa0ZaaSaaaeaacqGGBbWwcqWGpbWtcqGGOaakcqqGtbWudaWgaaWcbaGaemiEaGhabeaakiabcYcaSiabbofatnaaBaaaleaacqWG5bqEaeqaaOGaeiykaKIaeyOeI0IaemyrauKaeiikaGIaee4uam1aaSbaaSqaaiabdIha4bqabaGccqGGSaalcqqGtbWudaWgaaWcbaGaemyEaKhabeaakiabcMcaPiabc2faDnaaCaaaleqabaGaeGOmaidaaaGcbaGaemyrauKaeiikaGIaee4uam1aaSbaaSqaaiabdIha4bqabaGccqGGSaalcqqGtbWudaWgaaWcbaGaemyEaKhabeaakiabcMcaPaaaaaa@588C@

### Methods

For illustration, we will first give an exact algorithm D-MOTIF to find co-occurring motifs in *I*. Then, we will present our approximation algorithm, D-STAR, that can offer significant speed-up at the cost of slight accuracy degradation. The use of D-STAR for scaling up is necessary for dealing with the large input datasets in practice.

#### D-MOTIF algorithm

**Figure 5:**
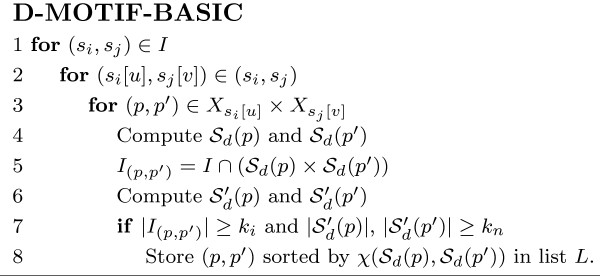
The D-MOTIF-BASIC algorithm.

The basic idea of the exact algorithm is to enumerate all possible (*l*, *d*)-motif pairs and then check if they have enough instances to satisfy the minimum size threshold *k*_*n*_and *k*_*i*_. Note that any (*l*, *d*)-motif pair must be of hamming distance *d *from some (*l*, *d*)-motif pair instance. Given a string *p *of length *l*, we define *X*_*p *_to be all strings *p' *of length *l *with hamming distances at most *d *from *p*. The algorithm named D-MOTIF-BASIC in Figure 5 describes the most straightforward brute force approach on the problem. Observe that the instances of any (*l*, *d*)-motif *x *would be of distance 2*d *from one another. Pevzner *et. al. *[17] described a method to compute all instances of an (*l*, *d*)-motif by transforming the problem into finding cliques in a *t*-partite graph *G*. In this graph, all *l*-substrings in all *s*_*i *_∈ *S *are the nodes and any two of them will be connected by an edge if (a) they originate from distinct proteins and (b) they are at most 2*d *apart. Thus, finding the (*l*, *d*)-motifs having at least *k*_*n *_instances is equivalent to finding cliques of size at least *k*_*n *_in *G*, which is an NP-hard problem.

We attempt to reduce the complexity of the problem by assuming that *k*_*n *_≥ 3 and try to find all cliques of size 3 first. In other words, we first find three *l*-substrings, (*s*_*i*_[*u*], *s*_*j*_[*v*], *s*_*k*_[*w*]), from distinct sequences *s*_*i*_, *s*_*j*_, and *s*_*k *_and then only try those candidate (*l*, *d*)-motifs *p *∈ Xsi[u]∩Xsj[v]∩Xsk[w]
 MathType@MTEF@5@5@+=feaafiart1ev1aaatCvAUfKttLearuWrP9MDH5MBPbIqV92AaeXatLxBI9gBaebbnrfifHhDYfgasaacH8akY=wiFfYdH8Gipec8Eeeu0xXdbba9frFj0=OqFfea0dXdd9vqai=hGuQ8kuc9pgc9s8qqaq=dirpe0xb9q8qiLsFr0=vr0=vr0dc8meaabaqaciaacaGaaeqabaqabeGadaaakeaacqWGybawdaWgaaWcbaGaem4Cam3aaSbaaWqaaiabdMgaPbqabaWccqGGBbWwcqWG1bqDcqGGDbqxaeqaaOGaeyykICSaemiwaG1aaSbaaSqaaiabdohaZnaaBaaameaacqWGQbGAaeqaaSGaei4waSLaemODayNaeiyxa0fabeaakiabgMIihlabdIfaynaaBaaaleaacqWGZbWCdaWgaaadbaGaem4AaSgabeaaliabcUfaBjabdEha3jabc2faDbqabaaaaa@4916@. For convenience, we call the string triplet (*s*_*i*_[*u*], *s*_*j*_[*v*], *s*_*k*_[*w*]) a *triangle *within *s*_*i*_, *s*_*j*_, and *s*_*k*_and we denote the set intersection Xsi[u]∩Xsj[v]∩Xsk[w]
 MathType@MTEF@5@5@+=feaafiart1ev1aaatCvAUfKttLearuWrP9MDH5MBPbIqV92AaeXatLxBI9gBaebbnrfifHhDYfgasaacH8akY=wiFfYdH8Gipec8Eeeu0xXdbba9frFj0=OqFfea0dXdd9vqai=hGuQ8kuc9pgc9s8qqaq=dirpe0xb9q8qiLsFr0=vr0=vr0dc8meaabaqaciaacaGaaeqabaqabeGadaaakeaacqWGybawdaWgaaWcbaGaem4Cam3aaSbaaWqaaiabdMgaPbqabaWccqGGBbWwcqWG1bqDcqGGDbqxaeqaaOGaeyykICSaemiwaG1aaSbaaSqaaiabdohaZnaaBaaameaacqWGQbGAaeqaaSGaei4waSLaemODayNaeiyxa0fabeaakiabgMIihlabdIfaynaaBaaaleaacqWGZbWCdaWgaaadbaGaem4AaSgabeaaliabcUfaBjabdEha3jabc2faDbqabaaaaa@4916@ by X(si[u],sj[v],sk[w])
 MathType@MTEF@5@5@+=feaafiart1ev1aaatCvAUfKttLearuWrP9MDH5MBPbIqV92AaeXatLxBI9gBaebbnrfifHhDYfgasaacH8akY=wiFfYdH8Gipec8Eeeu0xXdbba9frFj0=OqFfea0dXdd9vqai=hGuQ8kuc9pgc9s8qqaq=dirpe0xb9q8qiLsFr0=vr0=vr0dc8meaabaqaciaacaGaaeqabaqabeGadaaakeaacqWGybawdaWgaaWcbaGaeiikaGIaem4Cam3aaSbaaWqaaiabdMgaPbqabaWccqGGBbWwcqWG1bqDcqGGDbqxcqGGSaalcqWGZbWCdaWgaaadbaGaemOAaOgabeaaliabcUfaBjabdAha2jabc2faDjabcYcaSiabdohaZnaaBaaameaacqWGRbWAaeqaaSGaei4waSLaem4DaCNaeiyxa0LaeiykaKcabeaaaaa@466E@.

**Figure 6:**
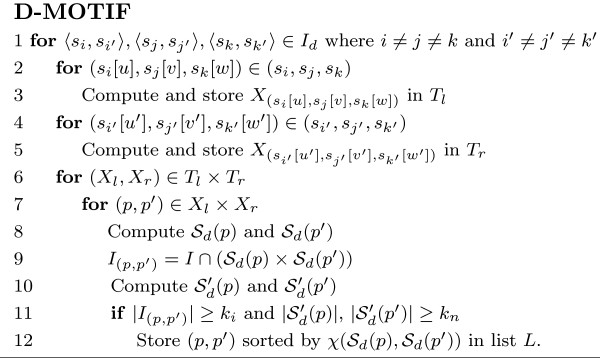
The D-MOTIF algorithm.

In the case of interaction data, we have to find all interaction triplets (*s*_*i*_*, s*_*i'*_), (*s*_*j*_, *s*_*j'*_), (*s*_*k*_, *s*_*k'*_) and compute the triangles from (*s*_*i*_*, s*_*j*_, *s*_*k*_) and (*s*_*i'*_, *s*_*j'*_, *s*_*k'*_). But as interaction is commutative (at least in our current consideration) i.e. (*s*_*i*_, *s*_*j*_) is equivalent to (*s*_*j*_, *s*_*i*_), we also have to consider the latter configuration when we choose the interaction triplets. As such, we let *I*_*d *_be the set of *ordered pair *which contains both ⟨*s*_*i*_, *s*_*j*_⟩ and ⟨*s*_*j*_, *s*_*i*_⟩ for each (*s*_*i*_, *s*_*j*_) ∈ *I*. The algorithm can then start by choosing the ordered pair triplets from *I*_*d*_(|*I*_*d*_| ≈ 2*m*). The complete listing of the algorithm, D-MOTIF, is presented in Figure 6.

In practice, D-MOTIF runs much faster when compared to the straightforward brute force algorithm(which we have also implemented as a benchmark). However, the memory requirement of D-MOTIF could be much larger than the latter as we have to store the sets *X *for the different triangles in the set *T*_*l *_and *T*_*r *_to avoid redundant computations. When *d *is large relative to *l*, there would be a lot of candidate (*l*, *d*)-motifs to check given a triangle. When the number of triangles is also large, even D-MOTIF would soon run at a crawling speed. In view of that, we propose the following approximation algorithm, D-STAR. Before we start, let us define the (*l*, *d*)-star pair finding problem and show how it approximates for the (*l*, *d*)-motif pair finding problem.

#### The (*l*, *d*)-star pair finding problem

For any given pair of *l*-substrings (*s*_*i*_[*u*], *s*_*j*_[*v*]) from some interaction (*s*_*i*_, *s*_*j*_), there may be an exponential (with respect to *d*) number of possible (*l*, *d*)-motifs (*x*, *y*) which is within distance *d*. Hence, even after speeding-up the algorithm with filtering, D-MOTIF can only handle relatively small-sized problems. In our proposed algorithm D-STAR, we will aim to find only the *instances *of a motif pair (*x*, *y*) instead of finding the motif (*x*, *y*) themselves since they may not even occur in *S*.

#### D-STAR algorithm

**Figure 7:**
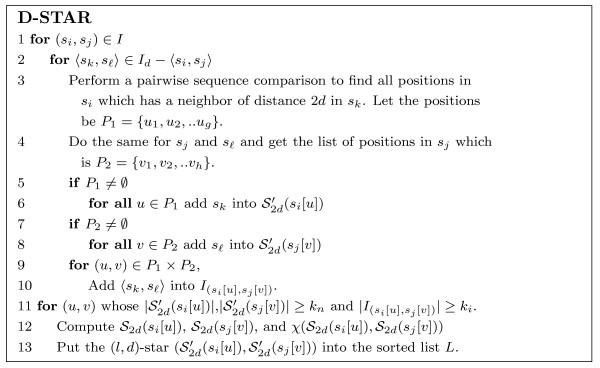
The D-STAR algorithm.

Recall that given an (*l*, *d*)-motif *x*, any two instances of *x*, *x*_*i *_and *x*_*j*_, would be at most 2*d *apart. Hence, if we manage to get one instance *x*_*i *_of *x*, all the other instances of *x *would surely be in S
 MathType@MTEF@5@5@+=feaafiart1ev1aaatCvAUfKttLearuWrP9MDH5MBPbIqV92AaeXatLxBI9gBamrtHrhAL1wy0L2yHvtyaeHbnfgDOvwBHrxAJfwnaebbnrfifHhDYfgasaacH8akY=wiFfYdH8Gipec8Eeeu0xXdbba9frFj0=OqFfea0dXdd9vqai=hGuQ8kuc9pgc9s8qqaq=dirpe0xb9q8qiLsFr0=vr0=vr0dc8meaabaqaciaacaGaaeqabaWaaeGaeaaakeaaimaacqWFse=uaaa@3845@_2*d*_(*x*_*i*_). In the context of interaction data, we first get all *l*-substring pairs (*s*_*i*_[*u*], *s*_*j*_[*v*]) from each interacting proteins (*s*_*i*_, *s*_*j*_) ∈ *I*. Next, we find those (*s*_*i*_[*u*], *s*_*j*_[*v*]) that satisfy two conditions (1) There are more than *k*_*i *_interactions between S
 MathType@MTEF@5@5@+=feaafiart1ev1aaatCvAUfKttLearuWrP9MDH5MBPbIqV92AaeXatLxBI9gBamrtHrhAL1wy0L2yHvtyaeHbnfgDOvwBHrxAJfwnaebbnrfifHhDYfgasaacH8akY=wiFfYdH8Gipec8Eeeu0xXdbba9frFj0=OqFfea0dXdd9vqai=hGuQ8kuc9pgc9s8qqaq=dirpe0xb9q8qiLsFr0=vr0=vr0dc8meaabaqaciaacaGaaeqabaWaaeGaeaaakeaaimaacqWFse=uaaa@3845@_2*d*_(*s*_*i*_[*u*]) and S
 MathType@MTEF@5@5@+=feaafiart1ev1aaatCvAUfKttLearuWrP9MDH5MBPbIqV92AaeXatLxBI9gBamrtHrhAL1wy0L2yHvtyaeHbnfgDOvwBHrxAJfwnaebbnrfifHhDYfgasaacH8akY=wiFfYdH8Gipec8Eeeu0xXdbba9frFj0=OqFfea0dXdd9vqai=hGuQ8kuc9pgc9s8qqaq=dirpe0xb9q8qiLsFr0=vr0=vr0dc8meaabaqaciaacaGaaeqabaWaaeGaeaaakeaaimaacqWFse=uaaa@3845@_2*d*_(*s*_*j*_[*v*]). (2) Let the set of the interactions be denoted similarly by I(si[u],sj[v])
 MathType@MTEF@5@5@+=feaafiart1ev1aaatCvAUfKttLearuWrP9MDH5MBPbIqV92AaeXatLxBI9gBaebbnrfifHhDYfgasaacH8akY=wiFfYdH8Gipec8Eeeu0xXdbba9frFj0=OqFfea0dXdd9vqai=hGuQ8kuc9pgc9s8qqaq=dirpe0xb9q8qiLsFr0=vr0=vr0dc8meaabaqaciaacaGaaeqabaqabeGadaaakeaacqWGjbqsdaWgaaWcbaGaeiikaGIaem4Cam3aaSbaaWqaaiabdMgaPbqabaWccqGGBbWwcqWG1bqDcqGGDbqxcqGGSaalcqWGZbWCdaWgaaadbaGaemOAaOgabeaaliabcUfaBjabdAha2jabc2faDjabcMcaPaqabaaaaa@3E73@, and we require that both |S′2d(si[u])|, |S′2d(sj[v])| ≥kn
 MathType@MTEF@5@5@+=feaafiart1ev1aaatCvAUfKttLearuWrP9MDH5MBPbIqV92AaeXatLxBI9gBamrtHrhAL1wy0L2yHvtyaeHbnfgDOvwBHrxAJfwnaebbnrfifHhDYfgasaacH8akY=wiFfYdH8Gipec8Eeeu0xXdbba9frFj0=OqFfea0dXdd9vqai=hGuQ8kuc9pgc9s8qqaq=dirpe0xb9q8qiLsFr0=vr0=vr0dc8meaabaqaciaacaGaaeqabaWaaeGaeaaakeaacqGG8baFimaacuWFse=ugaqbamaaBaaaleaaieaacqGFYaGmieGacqqFKbazaeqaaOGaeiikaGIaem4Cam3aaSbaaSqaaiabdMgaPbqabaGccqGGBbWwcqWG1bqDcqGGDbqxcqGGPaqkcqGG8baFcqGGSaalcqqGGaaicqGG8baFcuWFse=ugaqbamaaBaaaleaacqGFYaGmcqqFKbazaeqaaOGaeiikaGIaem4Cam3aaSbaaSqaaiabdQgaQbqabaGccqGGBbWwcqWG2bGDcqGGDbqxcqGGPaqkcqGG8baFcqqGGaaicqGHLjYScqWGRbWAdaWgaaWcbaGaemOBa4gabeaaaaa@5D90@. The pair of protein set (S′2d(si[u]), S′2d(sj[v])
 MathType@MTEF@5@5@+=feaafiart1ev1aaatCvAUfKttLearuWrP9MDH5MBPbIqV92AaeXatLxBI9gBamrtHrhAL1wy0L2yHvtyaeHbnfgDOvwBHrxAJfwnaebbnrfifHhDYfgasaacH8akY=wiFfYdH8Gipec8Eeeu0xXdbba9frFj0=OqFfea0dXdd9vqai=hGuQ8kuc9pgc9s8qqaq=dirpe0xb9q8qiLsFr0=vr0=vr0dc8meaabaqaciaacaGaaeqabaWaaeGaeaaakeaaimaacuWFse=ugaqbamaaBaaaleaaieaacqGFYaGmieGacqqFKbazaeqaaOGaeiikaGIaem4Cam3aaSbaaSqaaiabdMgaPbqabaGccqGGBbWwcqWG1bqDcqGGDbqxcqGGPaqkcqGGSaalcqqGGaaicuWFse=ugaqbamaaBaaaleaacqGFYaGmcqqFKbazaeqaaOGaeiikaGIaem4Cam3aaSbaaSqaaiabdQgaQbqabaGccqGGBbWwcqWG2bGDcqGGDbqxcqGGPaqkaaa@5213@) is denoted as an (*l*, *d*)-star pair. Our simulation experiments indicate that D-STAR yields a good approximation of the exact solution while being much more efficient when the dataset is large. The complete listing of the algorithm is in Figure 7.

#### Time complexity

The loop in line 1 takes *O*(*m*) time. The next loop in line 2 takes another *O*(*m*) time. Both pairwise sequence comparisons in step 3 and 4 require *O*(*n*^2^) time. Each time, the number of position pairs (*u*, *v*) in *P*_1 _× *P*_2 _could also reach *O*(*n*^2^). Updating I(si[u],sj[v])
 MathType@MTEF@5@5@+=feaafiart1ev1aaatCvAUfKttLearuWrP9MDH5MBPbIqV92AaeXatLxBI9gBaebbnrfifHhDYfgasaacH8akY=wiFfYdH8Gipec8Eeeu0xXdbba9frFj0=OqFfea0dXdd9vqai=hGuQ8kuc9pgc9s8qqaq=dirpe0xb9q8qiLsFr0=vr0=vr0dc8meaabaqaciaacaGaaeqabaqabeGadaaakeaacqWGjbqsdaWgaaWcbaGaeiikaGIaem4Cam3aaSbaaWqaaiabdMgaPbqabaWccqGGBbWwcqWG1bqDcqGGDbqxcqGGSaalcqWGZbWCdaWgaaadbaGaemOAaOgabeaaliabcUfaBjabdAha2jabc2faDjabcMcaPaqabaaaaa@3E73@, S′2d(si[u]), S′2d(sj[v])
 MathType@MTEF@5@5@+=feaafiart1ev1aaatCvAUfKttLearuWrP9MDH5MBPbIqV92AaeXatLxBI9gBamrtHrhAL1wy0L2yHvtyaeHbnfgDOvwBHrxAJfwnaebbnrfifHhDYfgasaacH8akY=wiFfYdH8Gipec8Eeeu0xXdbba9frFj0=OqFfea0dXdd9vqai=hGuQ8kuc9pgc9s8qqaq=dirpe0xb9q8qiLsFr0=vr0=vr0dc8meaabaqaciaacaGaaeqabaWaaeGaeaaakeaaimaacuWFse=ugaqbamaaBaaaleaaieaacqGFYaGmieGacqqFKbazaeqaaOGaeiikaGIaem4Cam3aaSbaaSqaaiabdMgaPbqabaGccqGGBbWwcqWG1bqDcqGGDbqxcqGGPaqkcqGGSaalcqqGGaaicuWFse=ugaqbamaaBaaaleaacqGFYaGmcqqFKbazaeqaaOGaeiikaGIaem4Cam3aaSbaaSqaaiabdQgaQbqabaGccqGGBbWwcqWG2bGDcqGGDbqxcqGGPaqkaaa@5213@, can all be done in constant time with a lookup table (one could save space using hash-sets, but the updating will take *amortized *constant time instead). The loop in line 11 would require at most *O*(*n*^2^) time for all entries [*u*, *v*], each requiring at most *O*(*t*) time to build (S2d(si[u]), S2d(sj[v]))
 MathType@MTEF@5@5@+=feaafiart1ev1aaatCvAUfKttLearuWrP9MDH5MBPbIqV92AaeXatLxBI9gBamrtHrhAL1wy0L2yHvtyaeHbnfgDOvwBHrxAJfwnaebbnrfifHhDYfgasaacH8akY=wiFfYdH8Gipec8Eeeu0xXdbba9frFj0=OqFfea0dXdd9vqai=hGuQ8kuc9pgc9s8qqaq=dirpe0xb9q8qiLsFr0=vr0=vr0dc8meaabaqaciaacaGaaeqabaWaaeGaeaaakeaacqGGOaakimaacqWFse=udaWgaaWcbaacbaGae4NmaidcbiGae0hzaqgabeaakiabcIcaOiabdohaZnaaBaaaleaacqWGPbqAaeqaaOGaei4waSLaemyDauNaeiyxa0LaeiykaKIaeiilaWIaeeiiaaIae8NeXp1aaSbaaSqaaiab+jdaYiab9rgaKbqabaGccqGGOaakcqWGZbWCdaWgaaWcbaGaemOAaOgabeaakiabcUfaBjabdAha2jabc2faDjabcMcaPiabcMcaPaaa@53AD@, from (S′2d(si[u]), S′2d(sj[v])
 MathType@MTEF@5@5@+=feaafiart1ev1aaatCvAUfKttLearuWrP9MDH5MBPbIqV92AaeXatLxBI9gBamrtHrhAL1wy0L2yHvtyaeHbnfgDOvwBHrxAJfwnaebbnrfifHhDYfgasaacH8akY=wiFfYdH8Gipec8Eeeu0xXdbba9frFj0=OqFfea0dXdd9vqai=hGuQ8kuc9pgc9s8qqaq=dirpe0xb9q8qiLsFr0=vr0=vr0dc8meaabaqaciaacaGaaeqabaWaaeGaeaaakeaaimaacuWFse=ugaqbamaaBaaaleaaieaacqGFYaGmieGacqqFKbazaeqaaOGaeiikaGIaem4Cam3aaSbaaSqaaiabdMgaPbqabaGccqGGBbWwcqWG1bqDcqGGDbqxcqGGPaqkcqGGSaalcqqGGaaicuWFse=ugaqbamaaBaaaleaacqGFYaGmcqqFKbazaeqaaOGaeiikaGIaem4Cam3aaSbaaSqaaiabdQgaQbqabaGccqGGBbWwcqWG2bGDcqGGDbqxcqGGPaqkaaa@5213@) for computing the χ-score. Therefore, in the worst case, D-STAR would run in *O*(*m*^2^*n*^2^+ *mtn*^2^). We also need to be mindful that the memory requirement to store the matrix and arrays is max{*O*(*mn*^2^), *O*(*tn*)}.

#### Comparison between D-MOTIF and D-STAR

First, we investigate the effect of data size on the performance of our two approaches. We ran our evaluation on 5 different datasets containing artificial interaction sets *I *of size ranging from 10 to 150 (note that for some weaker motifs, we did not evaluate up to size 150 as the running time of the D-MOTIF became too slow to be measured). In each interaction set, the protein sequences in all interaction are distinct; in other words, |*S*| = 2|*I*|. We also planted the (*l*, *d*)-motif pair in only half of the interactions in *I *to effect a fixed *ε *= 0.50 on all datasets.

**Figure 8:**
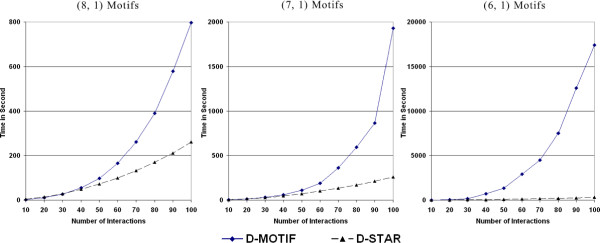
**Comparison of running time between D-MOTIF and D-STAR**. Observe that the running time of D-MOTIF increases rapidly as the input data grows and also as the (*l*, *d*)-motif gets weaker. All experiments were run on a x86 Pentium 4 1.6 GHz machine with 512 MB of memory.

Evaluation was performed here by checking if the planted motifs were reported as the *best *motif by the motif finding algorithm. Table 3 shows the average result over 10 datapoints (*I *= 10, 20, ..100) in each of the 5 evaluation datasets. Figure 8 displays the running time of both algorithms on different data size averaged over the 5 datasets. We use an x86 Pentium 4 M 1.6 GHz machine with 512 MB of memory for running the comparison. We observed that when the (*l*, *d*)-motifs get less specific and *k*_*n *_is small, the planted motifs could be masked out by other signals present in the protein sequences. This happened in one of the datapoints of (6, 1)-motifs with |*I*| = 10, in which D-STAR failed to have 100% sensitivity rate. Overall, it is clear that D-STAR performs only slightly worse than D-MOTIF while the running time of D-STAR is much better than D-MOTIF for larger datasets. The running time of D-MOTIF is also highly influenced by the strength/specificity of the (*l*, *d*)-motif. As compared to D-STAR, the running time of D-MOTIF increases much more rapidly when the motif gets less specific. For example, for |*I*| = 100, the running time of D-MOTIF on (8, 1), (7, 1), (6, 1) motifs are 797.4 s, 1930.7 s and 17385.2 s, respectively. For the same datapoints, D-STAR only required 253 s, 266.5 s, and 306.1 s, respectively. Indeed, this observation was further confirmed when we tried D-MOTIF on our real biological dataset later – it was still running after 10 hours while D-STAR terminates in less than 20 minutes.

**Table 3:** Comparison on specificity and sensitivity between D-MOTIF and D-STAR

(*l*, *d*)	D-MOTIF		D-STAR	
	
	Spec	Sens	Spec	Sens
(6, 1)	99.69%	100%	95.16%	99.1%
(7, 1)	100%	100%	99.89%	100%
(8, 1)	100%	100%	100%	100%

This table, in conjunction with Figure 8 showed that D-STAR runs orders of magnitude faster than D-MOTIF while sacrificing a small amount of accuracy in terms of sensitivity and specificity.

#### On choosing the parameters *k*_*n *_and *k*_*i*_

**Figure 9:**
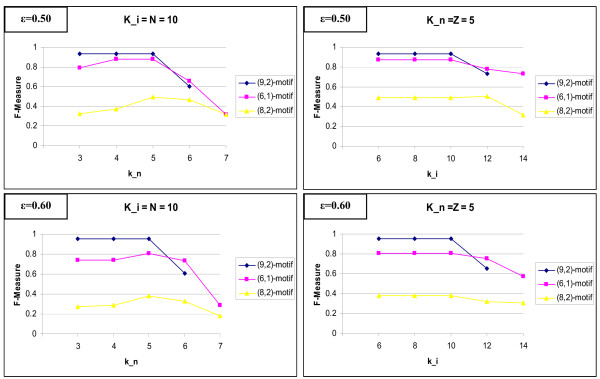
**Effect on varying *k*_*n *_and *k*_*i *_on the performance of D-STAR**. The experiments suggest that the nearer *k*_*n *_and *k*_*i *_to their actual value (the actual number of motif and motif pair in the dataset) would result in a better performance of the algorithm.

As with many other algorithm, the setting of the appropriate parameters would be a challenge for the user. Most of the time, these cannot be derived directly from the data. For D-STAR, one must set the minimum threshold parameters *k*_*n *_and *k*_*i *_for the minimum number of each motif instance and the minimum number of interaction that must be involved to derive the motifs. We performed a set of test where we vary the *k*_*n *_and *k*_*i *_value. The trend shows that the accuracy is highest when *k*_*n *_and *k*_*i *_is near their real value *Z*, where *Z *denotes the actual number of motif instances in the data, and *N*, the number of true interactions between the motif pair instances in the data, respectively. For strong motifs, accuracy is not affected even when *k*_*n *_or *k*_*i *_are set to relatively low values. For weaker motifs, it is easier to find spurious motifs and hence when *k*_*n *_and *k*_*i *_are too low, the performance will be poor. Hence we would suggest using large enough *k*_*n *_or *k*_*i *_and try to reduce them when one still cannot find any result. In general, like other existing motif algorithm, when the user has a good estimate of the length of the motif found, the quality of the motifs found would be better. The details can be found in Figure 9.

## Availability and requirements

Project name: Correlated motif discovery project.

Project homepage: 

Operating Systems: Windows XP, RedHat Linux, Solaris.

Programming Language: C.

License: The binaries used in the experiments are freely available in the website and in additional file 2.

## Authors' contributions

The project was initiated by SHT. The problem formulation was further polished and finalized by all authors. The algorithm design is done by WH, SHT and WKS. D-MOTIF and D-STAR were implemented by WH and all experiments was designed WKS, SHT and SKN, and were run by SHT. All authors contributed equally on the write-up and the analysis of the results obtained.

## Supplementary Material

Additional file 1 **SupplementaryData-DetailsOnMotifExtractedOnTGF-Beta**. The file contains detailed description on the motif instance set pair extracted by D-STAR and the corresponding known Phosphorylation motifs that is found enriched in one of the set. Click here for file

Additional file 2 **Program**. The file contains the binaries of the C implementation of D-STAR for Windows XP, Linux and Solaris OS. Also included in the zip file are the C2-BIND dataset as sample input and its sample output file. Click here for file
