# Supplementary material for: A correlated motif approach for finding short linear motifs from protein interaction networks
Source: BMC Bioinformatics. 2006 Nov 16;7:502. doi: 10.1186/1471-2105-7-502 (PMC1665647; doi:10.1186/1471-2105-7-502)
Supplement: Additional file 1 — SupplementaryData-DetailsOnMotifExtractedOnTGF-Beta. The file contains detailed description on the motif instance set pair extracted by D-STAR and the corresponding known Phosphorylation motifs that is found enriched in one of the set. [file 1471-2105-7-502-S1.pdf]

The Pair of Motif Instances Set extracted by D-STAR on the TGFβ Dataset

Kinase Protein Set		
**Proteins**	**Position**	**Segment**
GI:4502431	244	TEIYQT
GI:40254649	248	TELYNT
GI:4501895	248	TELYNT
GI:6678323	245	AEIYQT
GI:4759226	245	AEIYQT
GI:4885457	191	TETYST

The green-highlighted proteins are the proteins with real Kinase domains according to HMMER (5/6)

The red-highlighted proteins are the proteins with the phosphorylation sites as predicted by PhophoFinder (27/50)

Phophorylation Motifs Set		
**Proteins**	**Position**	**Segment**
GI:11024714	9	GKTITL
GI:11024714	85	GKTITL
GI:11024714	161	GKTITL
GI:11641237	20	GKTSII
GI:11967981	30	GKSSLA
GI:11967981	62	GATSLK
GI:13786127	73	SKRSLL
GI:13786129	44	GKTCLT
GI:16445426	149	GDTSLS
GI:19526471	85	GKTSRR
GI:19923750	33	GKTSFL
GI:21389385	17	GKTSLA
GI:22027525	769	SKTSIL
GI:30520350	27	GKTTIL
GI:34147073	199	LKNSLL
GI:41149704	277	GKRSTL
GI:41327767	32	GKTSLL
GI:4505571	262	GKRSRL
GI:4506713	9	GKTITL
GI:4506713	111	GKISRL
GI:4507449	28	GKTTFL
GI:4507761	9	GKTITL
GI:4757770	15	GKTSLL
GI:5031817	577	GCTSLK
GI:51036601	24	GKTSLI
GI:56243590	904	QKTPLL
GI:7656900	28	GKTSLL
GI:10835049	16	GKTCLL
GI:10864013	118	WKTALL
GI:12849714	113	QYTSLL
GI:13786127	47	GDTSFL
GI:16903164	32	GKTCLL
GI:16903164	65	GKQHLL
GI:21361884	17	GKSCLL
GI:22003858	176	GNTMLL
GI:22027525	280	EVTSLL
GI:22218619	357	GQGSLL
GI:24111250	290	NKTDLL
GI:24586657	784	GLLSLL
GI:30039692	363	GGGSLL
GI:31543537	63	GKTCLI
GI:4502741	272	GKDLLL
GI:4505451	47	GETCLL
GI:4505487	92	YGTSLL
GI:4506363	19	GKTCLI
GI:4506381	14	GKTCLL
GI:46249393	14	GKTCLL
GI:47717139	87	GKTMLN
GI:9966809	15	GKTAIL
GI:9966861	23	GKTNLL

Details on the Phosporylation Sites as Reported by PhophoFinder

Protein GI	Position	String	Site	Motif	Type
GI:11024714	9	GKTITL	KTIT	[R/K]xx[pS/pT]	PKC Kinase motif
GI:11024714	85	GKTITL	KTIT	[R/K]xx[pS/pT]	PKC Kinase motif
GI:11024714	161	GKTITL	KTIT	[R/K]xx[pS/pT]	PKC Kinase motif
GI:11024714	9	GKTITL	KTIT	Kxx[pS/pT]	PKA Kinase motif
GI:11024714	85	GKTITL	KTIT	Kxx[pS/pT]	PKA Kinase motif
GI:11024714	161	GKTITL	KTIT	Kxx[pS/pT]	PKA Kinase motif
GI:11641237	20	GKTSII	KTS	[R/K]x[pS/pT]	PKC and PKA Kinase motif
GI:11967981	62	GATSLK	SLK	[pS/pT]x[R/K]	PKC and PKA Kinase motif
GI:11967981	30	GKSSLA	KSS	[R/K]x[pS/pT]	PKC and PKA Kinase motif
GI:13786127	73	SKRSLL	SKR	[pS/pT]x[R/K]	PKC and PKA Kinase motif
GI:13786127	73	SKRSLL	SKRS	[pS/pT]xx[S/T/Y]	CK2 Kinase motif
GI:13786127	73	SKRSLL	SKRS	[pS/pT]xxS	CK1 Kinase motif
GI:13786127	73	SKRSLL	KRS	[R/K]x[pS/pT]	PKC and PKA Kinase motif
GI:13786127	73	SKRSLL	SKRS	pSxx[E/pS/pT]	CK2 and Casein Kinase motif
GI:13786129	44	GKTCLT	TCLT	[pS/pT]xx[S/T/Y]	CK2 Kinase motif
GI:13786129	44	GKTCLT	KTCLT	Kxxx[pS/pT]	PKA Kinase motif
GI:16445426	149	GDTSLS	TSLS	[pS/pT]xx[S/T/Y]	CK2 Kinase motif
GI:16445426	149	GDTSLS	TSLS	[pS/pT]xxS	CK1 Kinase motif
GI:19526471	85	GKTSRR	TSR	[pS/pT]x[R/K]	PKC and PKA Kinase motif
GI:19526471	85	GKTSRR	KTS	[R/K]x[pS/pT]	PKC and PKA Kinase motif
GI:19526471	85	GKTSRR	KTSRR	[R/K]x[pS/pT]x[R/K]	PKC Kinase motif
GI:19923750	33	GKTSFL	KTS	[R/K]x[pS/pT]	PKC and PKA Kinase motif
GI:21389385	17	GKTSLA	KTS	[R/K]x[pS/pT]	PKC and PKA Kinase motif
GI:22027525	769	SKTSIL	SKTS	[pS/pT]xx[S/T/Y]	CK2 Kinase motif
GI:22027525	769	SKTSIL	SKTS	[pS/pT]xxS	CK1 Kinase motif
GI:22027525	769	SKTSIL	KTS	[R/K]x[pS/pT]	PKC and PKA Kinase motif
GI:22027525	769	SKTSIL	SKTS	pSxx[E/pS/pT]	CK2 and Casein Kinase motif
GI:30520350	27	GKTTIL	KTT	[R/K]x[pS/pT]	PKC and PKA Kinase motif
GI:34147073	199	LKNSLL	KNS	[R/K]x[pS/pT]	PKC and PKA Kinase motif
GI:41149704	277	GKRSTL	KRST	[R/K][R/K]x[pS/pT]	PKA Kinase motif
GI:41149704	277	GKRSTL	KRST	[R/K][R/x]x[pS/pT]	PAKs phosphorylation motif
GI:41149704	277	GKRSTL	KRS	[R/K]x[pS/pT]	PKC and PKA Kinase motif
GI:41149704	277	GKRSTL	KRST	[R/K]xx[pS/pT]	PKC Kinase motif
GI:41149704	277	GKRSTL	KRST	Kxx[pS/pT]	PKA Kinase motif
GI:41327767	32	GKTSLL	KTS	[R/K]x[pS/pT]	PKC Kinase motif
GI:4505571	262	GKRSRL	KRS	[R/K]x[pS/pT]	PKC and PKA Kinase motif
GI:4506713	111	GKISRL	KIS	[R/K]x[pS/pT]	PKC and PKA Kinase motif
GI:4506713	9	GKTITL	KTIT	[R/K]xx[pS/pT]	PKC Kinase motif
GI:4506713	9	GKTITL	KTIT	Kxx[pS/pT]	PKA Kinase motif
GI:4507449	28	GKTTFL	KTT	[R/K]x[pS/pT]	PKC and PKA Kinase motif
GI:4507761	9	GKTITL	KTIT	[R/K]xx[pS/pT]	PKC Kinase motif
GI:4507761	9	GKTITL	KTIT	Kxx[pS/pT]	PKA Kinase motif
GI:4757770	15	GKTSLL	KTS	[R/K]x[pS/pT]	PKC and PKA Kinase motif
GI:5031817	577	GCTSLK	SLK	[pS/pT]x[R/K]	PKC and PKA Kinase motif
GI:51036601	24	GKTSLI	KTS	[R/K]x[pS/pT]	PKC and PKA Kinase motif
GI:56243590	904	QKTPLL	TP	[pS/pT]P	Proline-directed Kinase motif
GI:56243590	904	QKTPLL	KTP	[R/K][pS/pT]P	Growth-associated histone HI Kinase motif
GI:7656900	28	GKTSLL	KTS	[R/K]x[pS/pT]	PKC and PKA Kinase motif
